# 506 Review of Empiric Voriconazole Dosing in Large TBSA Burn Patients – A Case Series

**DOI:** 10.1093/jbcr/irad045.103

**Published:** 2023-05-15

**Authors:** Lauren Lautenslager, Allison Boyd, Brett Hartman, Leigh Spera

**Affiliations:** Indiana University School of Medicine, Indianapolis, Indiana; Eskenazi Health, Indianapolis, Indiana; Indiana university/Eskenazi Health, Indianapolis, Indiana; Indiana University School of Medicine/Eskenazi Health, Indianapolis, Indiana; Indiana University School of Medicine, Indianapolis, Indiana; Eskenazi Health, Indianapolis, Indiana; Indiana university/Eskenazi Health, Indianapolis, Indiana; Indiana University School of Medicine/Eskenazi Health, Indianapolis, Indiana; Indiana University School of Medicine, Indianapolis, Indiana; Eskenazi Health, Indianapolis, Indiana; Indiana university/Eskenazi Health, Indianapolis, Indiana; Indiana University School of Medicine/Eskenazi Health, Indianapolis, Indiana; Indiana University School of Medicine, Indianapolis, Indiana; Eskenazi Health, Indianapolis, Indiana; Indiana university/Eskenazi Health, Indianapolis, Indiana; Indiana University School of Medicine/Eskenazi Health, Indianapolis, Indiana

## Abstract

**Introduction:**

Burn patients are significantly immunocompromised and susceptible to opportunistic fungal infections. Treatment includes aggressive surgical debridement with topical and systemic anti-fungal agents. Voriconazole (VCZ) is a systemic anti-fungal and an approved first line treatment of invasive Aspergillus and Fusarium species. Standard dosing is a 6 mg/kg loading dose twice followed by 200 mg enterally twice daily. Therapeutic drug monitoring is recommended to confirm a goal trough concentration of >1 mcg/mL, once steady state is reached, approximately 4-7 days after initiation. At our institution, VCZ levels are a send out lab, averaging 4-7 days for results. We reviewed a series of three patients with invasive fungal infections and their VCZ treatment dosing to assess impact of altered pharmacokinetics and time delays with drug monitoring.

**Methods:**

Three patients with significant burn injury (TBSA >40%) and invasive fungal wound infections were reviewed. Two were treated with standard dosing of VCZ (200 mg BID). The %TBSA, weight, albumin at time of VCZ initiation, initial VCZ dosing, initial trough concentration, time from initiation to trough result and final VCZ dose was reviewed. Consideration of the subtherapeutic drug level of prior patients led to initiation of VCZ treatment at an increased initial empiric dose (300 mg BID) for a third patient, followed by assessment and review of the same variables.

**Results:**

For the two patients treated with standard VCZ dosing, initial trough concentrations were subtherapeutic (0.4 mcg/mL and 0.9mcg/mL) and took 13 and 10 days from initiation to trough result, respectively. The albumin concentrations for these patients were 2.1 and 1.6 g/dL. Initial trough concentration for the third patient started on increased empiric dosing was therapeutic (4.9 mcg/mL), despite an albumin of < 1 g/dL. (Table 1).

**Conclusions:**

Fungal infections significantly increase the morbidity and mortality of burn patients. The time lapse from initial dose to steady state plus turnaround time of send out labs may result in 1-2 weeks of subtherapeutic treatment. Results from our case series demonstrate that standard VCZ dosing could be inadequate for large TBSA burn patients ( >40%) and higher empiric doses should be considered.

**Applicability of Research to Practice:**

Consider use of increased initial VCZ dosing for large burn patients to reach therapeutic serum levels more expeditiously.

